# Oxidant/Antioxidant Balance in Animal Nutrition and Health: The Role of Protein Oxidation

**DOI:** 10.3389/fvets.2015.00048

**Published:** 2015-10-26

**Authors:** Pietro Celi, Gianfranco Gabai

**Affiliations:** ^1^DSM Nutritional Products, Animal Nutrition and Health, Columbia, MD, USA; ^2^Faculty of Veterinary and Agricultural Sciences, The University of Melbourne, Parkville, VIC, Australia; ^3^Department of Comparative Biomedicine and Food Science, University of Padova, Legnaro, Italy

**Keywords:** protein oxidation, biomarkers, oxidative stress, inflammation, animal welfare

## Abstract

This review examines the role that oxidative stress (OS), and protein oxidation in particular, plays in nutrition, metabolism, and health of farm animals. The route by which redox homeostasis is involved in some important physiological functions and the implications of the impairment of oxidative status on animal health and diseases is also examined. Proteins have various and, at the same time, unique biological functions and their oxidation can result in structural changes and various functional modifications. Protein oxidation seems to be involved in pathological conditions, such as respiratory diseases and parasitic infection; however, some studies also suggest that protein oxidation plays a crucial role in the regulation of important physiological functions, such as reproduction, nutrition, metabolism, lactation, gut health, and neonatal physiology. As the characterization of the mechanisms by which OS may influence metabolism and health is attracting considerable scientific interest, the aim of this review is to present veterinary scientists and clinicians with various aspects of oxidative damage to proteins.

## Introduction

In aerobic organisms, mitochondrial and cytochrome P450 metabolism, inflammation processes (e.g., phagocyte respiratory burst) and several environmental factors are endogenous sources of reactive oxygen species (ROS) (1). In low concentrations, ROS are involved in numerous physiological events (2, 3), but they can be detrimental for living cells if they are present in excessive amounts (4) that cannot be overridden by the body antioxidants. The imbalance between pro-oxidants and antioxidants lead to oxidative stress (OS), eventually resulting in damages of macromolecules.

**Figure 1:**
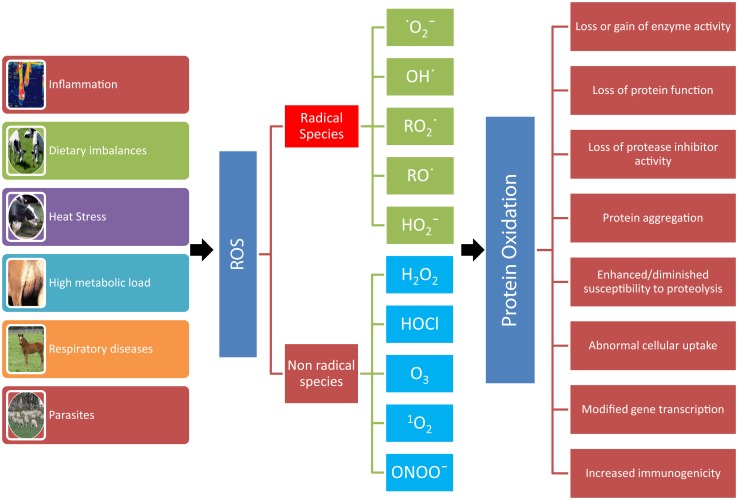
**Consequences of protein oxidation**. A number of conditions (inflammation, dietary imbalances, heat stress, high metabolic load, respiratory diseases, and parasites) can lead to the formation of reactive oxygen metabolites (ROS). ROS leading to protein oxidation that includes radical species, such as superoxide $\left({\;}^{•}{\text{O}}_{2}^{-}\right)$, hydroxyl (OH^•^), peroxyl $\left({\text{RO}}_{\text{2}}^{•}\right)$, alkoxyl (RO^•^), hydroperoxyl $\left({\text{HO}}_{2}^{-}\right)$, and non-radical species, such as hydrogen peroxide (H_2_O_2_), hypochlorous acid (HOCl), ozone (O_3_), singlet oxygen (^1^O_2_), and peroxynitrite (ONOO^−^). The oxidation of protein can result in loss or gain of enzyme activity, loss of protein function and of protease inhibitor activity, protein aggregation, enhanced/diminished susceptibility to proteolysis, abnormal cellular uptake, modified gene transcription, and increased immunogenicity.

The role of OS in farm animals’ health and disease has been reviewed elsewhere (5–7). The most investigated causes of OS in veterinary medicine are metabolic and inflammatory events, and environmental factors (heat stress and nutrition) (Figure 1). In dairy cows, conditions, such as high body condition score at calving (8), high milk yield (9), negative energy balance (10) and diet (11), and suppressed dry period (12) are all factors contributing to increased OS. Several observations suggest that excessive lipid mobilization plays a pivotal role as a link between altered energy metabolism, OS and decrease immune system efficiency (13, 14). Indeed, the adipose tissue secretes a great number of substances involved in the modulation of the immune response (5, 15), and during excessive adipose tissue mobilization, adipose tissue produces proinflammatory cytokines, while the production of adiponectin is reduced (16). In addition, changes in bovine NEFA concentrations and composition may alter the response of monocyte and neutrophil (17).

Activated phagocytes and neutrophils represent another source of OS, which contribute to OS by generating hydrogen peroxide and superoxide via a respiratory burst, and the release of the enzyme myeloperoxidase (MPO), which catalyzes the reaction of hydrogen peroxide with physiological concentrations of chlorine ions and gives rise to the potent oxidant hypochlorous acid. Hypochlorous acid plays an important role in killing invading pathogens; however, excessive or misplaced generation of this oxidant can cause tissue damage, and it is believed to be involved in a number of human and animal diseases (18).

Oxidative stress can virtually damage all biological molecules (DNA, RNA, cholesterol, lipids, carbohydrates, and proteins). In turn, the oxidation of these macromolecules produces various endproducts that can be measured to assess OS *in vivo*. Proteins are the molecules most susceptible to oxidative damage in cells because they are often catalysts rather than stoichiometric mediators; hence, the effect of damage to one molecule is greater than stoichiometric (19). Proteins have various and unique biological functions, and their oxidation can result in structural changes and consequently in various modifications in their functions (Figure 1) (20–22).

The study of the physiopathology of protein oxidation has attracted considerable interest in human medicine (Table 1), but similar studies in veterinary medicine are still scarce. Therefore, the aim of this review is to present veterinary scientists and clinicians with various aspects of oxidative damage to proteins, with emphasis on using oxidized proteins as markers of OS in veterinary medicine. We will also discuss the involvement of protein oxidation in pathological conditions relevant to farm animals. In this regard, we have used the modern dairy cow as it has been the objective of the majority of studies conducted in this field; examples of other species have been given when available. Besides oxygen, nitrogen also plays an important role in damaging biological molecules via “reactive nitrogen species.” However, this review will not deal with this family of reactive species.

**Table 1:** **Approximate constitution of various biological materials and number of papers published in PubMed related to protein, DNA, and lipid oxidation [adapted from Ref. (23)]**.

	Protein	DNA	Total lipid	Cholesterol
Organ level (liver, g/kg wet weight)	146	2.6	49	3.9
Cellular level (g/10^12^ leukocytes)	100	6.9	15.6	2
Plasma (g/L)	73		0.5	1.5–2.5
Papers (PubMed)	106,508	19,561	55,287	7,394

## Protein Oxidation

It has been estimated that proteins can scavenge up to 75% of free radicals, such as hydroxyl (24). On the basis of this evidence and considering that oxidized proteins have considerably long half-lives, it is reasonable to assume that proteins are likely to accumulate “evidence” of oxidative insult making those suitable markers of oxidative damage (23, 25).

Protein oxidation is defined as the covalent modification of a protein induced either directly by ROS or indirectly by reaction with secondary by-products of OS (22). Oxidative damage to proteins can affect their functions as receptors, enzymes, transport, or structural proteins, etc.; moreover, oxidized proteins can generate new antigens and provoke immune response (26).

Reactive species leading to protein oxidation are outlined in Figure 1 and include radical and non-radical species (21, 25). Other agents that lead to protein oxidation include reagents, such as xenobiotics, such as paraquat, CCl_4_, and acetaminophen, cigarette smoke, reduced transition metals, such as Fe^2+^ or Cu^+^, γ-irradiation in the presence of O_2_, activated neutrophils, ultraviolet light, ozone, oxidoreductase enzymes, and by-products of lipid and free amino acid oxidation (21, 25). For example, pigs exposed to whole body γ-irradiation showed a significant increase in protein carbonyls (27). As a consequence of the large variety of agents that cause protein oxidation and because all of the amino acyl side chains can become oxidatively modified, there are numerous different types of protein oxidative modification (22).

Reactions of ROS with proteins and peptides can induce alterations to both the backbone and side chains (25). The use of backbone fragmentation as a marker of protein oxidation *in vivo* is very limited because of the quantity of other proteins present and the potential role of proteases that can bias the results (28). Nevertheless, the use of protein fragmentation as a potential source of biomarkers is very attractive in veterinary science, and mastitis detection seems a promising field of investigation. During mastitis infection, pathogenic bacteria elude the defense mechanisms of the mammary gland, start multiplying and liberate enzymes and toxins. Circulating polymorphonuclear neutrophils (PMNs) are attracted to the site of infection and release oxidants and proteases to destroy the invading pathogens (29). Enzymes and oxidants secreted by both bacteria and PMNs result in the damage of the mammary cells and milk components, milk proteins in particular. Interestingly, the peptidome resulting from the fragmentation of milk proteins in diseased animals shows peculiar differences from that observed in healthy animals, and it may be possible to obtain a panel of protein fragments, which can be used for differential diagnosis of causative bacteria of mammary infection (29).

Products of protein side chain oxidation, on the other hand, are relatively stable and there are several assays available for their detection (30). The most frequently used biomarker of protein oxidation is the carbonyl assay, which measures protein carbonyl groups (26, 30). Protein carbonylation occurs when ROS attack the amino acid side chains of proline, arginine, lysine, and threonine in presence of transition metals (Fe^2+^, Cu^+^, etc.). This process can be defined as “primary protein carbonylation,” and refers to the formation of reactive ketones or aldehydes that can react with 2,4-dinitrophenylhydrazine (DNPH) (31). Alternatively, reactive carbonyl compounds, such as carbohydrates and lipids having reactive carbonyl groups produced during glycoxidation and lipoperoxidation, can bind to amino acid side chains (mainly on lysine, cysteine, and histidine residues) generating the so-called “secondary protein carbonylation” (31, 32).

Protein carbonylation is the most frequent irreversible transformation and also the one most often studied (30), and accumulation of protein carbonyls has been observed in several human diseases (21, 33, 34). In general, the accumulation of oxidized proteins depends on the rate of their clearance. Degradation of oxidized proteins is influenced by the presence and the activity of specific proteases and the extent of their chemical modification, with mildly oxidized proteins highly prone to degradation, while extremely oxidized proteins (i.e., carbonylated) can form cross-links and aggregates, which are poor substrates for proteolysis (35). Therefore, these molecules are chemically stable, which is advantageous for both their detection and storage, with several assays being available for their detection.

Carbonyls have no specific UV or visible spectrophotometric absorbance/fluorescence properties; therefore, it is not possible to detect them directly. Products of both primary and secondary carbonylation react with chemical probes, such as DNPH, which allows the determination of total protein carbonyl groups. Once a protein carbonyl reacts with DNPH, a 2,4-dinitrophenylhydrazone (DNP)-protein adduct is formed, and total carbonyl groups in a mixture of proteins can be detected and quantified spectrophotometrically due to the characteristic absorption spectrum of DNP with a maximum at 365–375 nm. Moreover, as several antibodies against DNP are commercially available, carbonyl groups can be detected also by immunoassays, such as ELISA and Western blot (36).

Proteins can be oxidized in more than 35 ways, and many of these modifications involve some form of carbonylation (37), thus the carbonyl assay applied to tissues or body fluids measures the average protein modifications (26). However, total carbonyl determination does not provide accurate information on the source of the OS insult, as in most cases, the carbonylated protein(s) cannot be identified. Moreover, an inaccurate estimation of carbonyl groups can occur when they are measured spectrophotometrically using DNPH as the chemical probe, because some proteins (e.g., hemoglobin, cytochrome c) have absorbance wavelengths similar to DNPH (38).

In the last decade, the development of mass spectrometry (MS) methods, coupled with suitable protein fractionation strategies, have been developed. Comprehensive reviews of MS methods and enrichment strategies to study post-translational protein modifications have been recently published (36, 39). Here, it is worth remembering that combining the derivatization of carbonyl groups with reagents, such as DNPH with proteomic techniques, leads to the identifications of specific carbonylated proteins in several diseases in humans, animal models, and cell models (31, 36, 37). Indeed, proteomic approaches suggest that not all the proteins in a given proteome are subjected to the same oxidative attack, and carbonyl accumulation during OS, disease, or aging is a selective process (40).

Proteins rich in cysteine and methionine residues, both of which contain susceptible sulfur atoms, are particularly prone to OS damage. In the case of cysteine, oxidation leads to the formation of disulfide bonds, mixed disulfides, and thiyl radicals, while methionine sulfoxide is the major product of methionine oxidation (22, 28). The oxidation of cysteine and methionine is reversible as cells are equipped with systems, such as methonine sulfoxide reductase, glutathione, and thioredoxin redox system, capable of reversing the oxidation. It seems that the reversible oxidation/reduction of methionine may prevent the formation of more damaging forms of protein oxidative modification, namely protein carbonyl formation (22). Oxidation of cysteine and methionine can also result in the reversible formation of disulfides bonds between thiol groups (41). Detection of reversible oxidative modifications, however, cannot be considered as useful biomarkers of protein oxidation.

Hypochlorous acid-induced products are another major group of protein oxidation products. Primary products are chloro- and di-tyrosyl residues, amino acyl aldehyde adducts, and chloramines. They represent unique products of MPO activity, reflecting neutrophil and monocyte activity and therefore they can serve as indicators of OS markers generated during the inflammatory response (22). The availability of biomarkers for HOCl is important for investigating the pathological role of MPO and its products.

Dityrosine is a fluorescent molecule that represents one of the normal post-translational modifications of proteins, which implies a protein-protein cross-linking through tyrosine-tyrosine binding. In addition, dityrosine can be found as the product of oxidative or nitrative stress under a number of disease conditions, such as in the atherosclerotic plaque, Alzheimer’s brain tissue, human blood plasma, and urine of humans and animals (42, 43). The ionized form of dityrosine have maximum absorption at 315 nm (44) and it is characterized by an intense fluorescence at 420 nm measurable upon excitation at either 315 nm (alkaline solution) or 284 nm (acidic solution) (43). Dityrosine is widely used as an important biomarker of oxidative modified proteins as it is a stable product and it is released by proteolysis of proteins modified by OS. Moreover, free dityrosine cannot be incorporated into proteins synthesized “*de novo*,” thus suggesting that the levels of dityrosine reflect the oxidative damage of proteins (43).

Analytical methods to detect and measure dityrosine have been extensively reviewed elsewhere (42–44). The simplest way to monitor dityrosine formation “*in vitro*” is measuring the absorbance at 315 nm in a pure protein solution (45); however, spectrophotometric measurement of dityrosine cannot be used in a complex matrix, such as serum/plasma, as many chromophores absorb in the same wavelength interval. Dityrosine residues quantification can be performed in plasma samples by a fluorimetric method, after the sample is denaturated by dilution in PBS with 6 M urea (46, 47). The fluorescence emission is read near 410 nm after excitation at 325 nm, and the assay is calibrated using authentic synthesized dityrosine (48).

Dityrosine in tissues can be visualized by immunohistochemical staining using one of the commercially available antibodies. The availability of antibodies specific for dityrosine enabled also the development of immunoassays and Western blot analysis of solubilized protein extracts (43). However, the quantitative determination of dityrosine released by oxidized proteins usually employs the combinations of chromatographic techniques (42–44). Recently, quantitative measurement of dityrosine can be performed by MS, and the molecule has been detected in biological matrices of veterinary interest, such as bovine milk protein (49), cat urine (50), and rat plasma (51).

Dityrosine is a product of MPO and activated bovine neutrophils can produce a significant amount of dityrosine from bovine serum albumin (45). When proteins containing tyrosyl residues are exposed to HOCl, other compounds, such as 3-chlorotyrosin and 3,5-dichlorotyrosine are formed, but sensitive and complex procedures are required to detect tyrosine chlorination after exposure to physiological amounts of HOCl (18).

Advanced oxidation protein products (AOPP) can be defined as synthetic markers of protein oxidation. Spectral characteristics of AOPP correspond to chromophores induced by chlorinated oxidants, such as 3-chlorotyrosin and 3,5-dichlorotyrosine (52). Moreover, AOPP contain abundant dityrosine and disulfide bridges, which allow cross-linking, and carbonyl groups (46, 53). AOPP increase as a result of neutrophil activation during infections as they represent useful markers of MPO/HOCl protein oxidation. Conversely, although protein carbonyls can arise through the breakdown of chloramines, they cannot be taken as being specific biomarkers for HOCl (18) as a variety of other oxidation mechanisms can lead to their formation (25).

When measuring AOPP in plasma by the original method developed by Witko-Sarsat et al. (46), it should be considered that AOPP comprises several chromophores (46, 52, 53). In humans, fibrinogen (54) and serum albumin (46) modifications, and dityrosine cross-links are the major contributors to AOPP formation. Indeed, AOPP measurement can be used to estimate the dityrosine content in a biological matrix (54). The original assay (46), however, could lead to an overestimation of AOPP, particularly due to the influence of plasma turbidity induced by triglycerides (55). For this reason, a modified AOPP assay, which included a sample preparation procedure to precipitate plasma lipoproteins, has been developed (56). More recently, a method using citric acid to solubilize plasma lipids was developed in order to overcome the reported overestimation of AOPP levels (57). This method should allow a more accurate measure of chromophore absorption at 340 nm. However, AOPP analysis based on absorbance of light at 340 nm is not a selective way to measure oxidized proteins, and we consider of the utmost importance to test the potential interference of lipids or other substances when applying the AOPP assay to a new animal species or to a different biological fluid.

## Diseases and Protein Oxidation in Farm Animals

### Protein Oxidation and Female Reproduction

It is apparent that OS plays a crucial role in the cause and progression of a number of reproductive events, such as fertilization and early embryo development (58), and indeed it seems that OS is involved in the regulation of the female reproductive system at different levels (7, 59). For example, it has been shown that OS is associated with embryonic losses in dairy cows (60, 61) and that OS is involved in the pathogenesis of follicular cysts and repeat breeder syndrome in dairy cows (62–64).

It has been observed that the plasma profiles of the AOPP/albumin ratio provide a more sensitive indicator of OS (60, 61). This observation supports the proposed development and validation of a protein oxidation index. Considering that in dairy cows, pathogens are often introduced in the uterus during the artificial insemination procedure, it could be argued that they could generate an inflammatory reaction leading to OS and AOPP generation. Indeed, the development of subclinical endometritis seems to be a common event in dairy cows after artificial insemination (65), therefore the increase in plasma AOPP in cows that experience embryonic mortality might be indicative of subclinical uterine infection. It is important to consider that when uterine physical defenses are breached, the next line of defense is represented by neutrophils and macrophages triggering an inflammation process: the resulting activated leukocyte and vasoactive substances released during inflammation can increase blood vessel permeability resulting in plasma protein leaking into the endometrial surface (66, 67). We, therefore, propose that the assessment of OS and protein oxidation in particular, might improve our understanding of the role of OS and protein oxidation in the pathophysiology of reproductive wastage.

As consequence of the rapid fetal growth during the last trimester of pregnancy and the production of large amounts of colostrum and milk at the beginning of lactation, both maternal and fetal metabolism is increased in consequence of augmented mitochondrial activity in maternal tissues and the conceptus (68). This results in an increase in the production of ROS, particularly in dairy cows during late gestation (69) and early lactation (10). Considering that the activity of monocytes and macrophages is increased during pregnancy, and that the concentration of several markers of OS is concomitantly increased, it could be argued that pregnancy is characterized by a proinflammatory state (70). The observation of a positive correlation between AOPP and C-reactive protein during pregnancy brings further support to the association between inflammation and OS during pregnancy (71).

Studies in sows have reported a close relationship between protein oxidation and reproductive performance, with negative correlations between plasma protein carbonyl and litter size and litter weights (72). It is possible that the observed increase in protein oxidation and consequent increase in functional modification of proteins, protein turnover, and cell death, as discussed above, might be responsible for the observed decrease in litter size in sows in high social rank treatment (72).

One possible approach to reduce OS during late gestation is given by antioxidant supplementation. Studies in pigs have been able to demonstrate that silymarin supplementation of gilts’ diets decreased liver and circulating protein carbonyl (73). Conversely, the administration of polyphenols rich foods (dried tomatoes, dried apples, dried green tea leaves, and raw soy grains) in pregnant ewes resulted in an increase in protein carbonyls and a decrease in lipid peroxidation and non-protein thiols (74). Considering the complexity of numerous interactions between antioxidants and body systems (genome, proteome, and metabolome), it is conceivable that a thorough analysis of antioxidants–animal interactions is necessary to achieve a deeper understanding of the effects of antioxidant supplementation in animal diets. The use of plants and plant rich in antioxidants is a new goal in animal nutrition (75–77), which needs to be explored.

### Protein Oxidation, Nutrition, and Gut Health

In dairy cattle, prolonged concentrate feeding increased lipid peroxidation, and decreased α-tocopherol and ferric reducing ability of plasma (78). An increase in OS has also been observed when high levels of starch have been fed to dairy cows (11). It has been demonstrated that an increase in dietary concentrate content and a reduction in dietary NDF content are associated with an increase in ruminal endotoxin (79), which may stimulate the production of proinflammatory cytokines, ROS, and bioactive lipids (80). When ruminal endotoxin and plasma OS biomarker concentrations were evaluated in dairy heifers fed with grain, fructose, and histidine, and their combinations under subacute ruminal acidosis (SARA) challenge conditions, no effects of dietary treatments were observed on OS biomarkers (81). Considering the observed decreases in ruminal pH, increases in total VFA, and marked increases in lactic acid in fructose-fed heifers (82), changes in OS biomarkers were expected; however, the lack of OS response including protein oxidation (AOPP) to the dietary treatments could be ascribed to the lack of treatment effects on ruminal endotoxin (81), which is consistent with the hypothesis that AOPP increase early during the inflammatory pathway, when PMN cells are recruited and activated. The absence of effects on AOPP may also be indicative of the acute, as opposed to the chronic challenge of the study. In support of this hypothesis are the observations made in growing lambs that were fed a pelleted diet for 8 weeks, where a significant increase in concentrate intake was paralleled by an increase in AOPP concentration in lambs supplemented with Yerba Mate (83). Considering that high level of feed intake are negatively associated with rumen buffering capacity (84), it is likely that the higher levels of concentrate intake in the Yerba Mate supplemented lambs might have caused a transient state of SARA and that the observed higher levels of AOPP may reflect subclinical inflammatory events. Further research is needed to explore the relationships between abruptly feeding rapidly fermentable carbohydrates, endotoxin, and OS and to determine thresholds for OS induced changes in metabolism. In this context, plasma concentration of AOPP may be indicative of an early inflammatory event. Considering that AOPP are very easy to measure, it seems worthwhile exploring the possible applications of using this biomarker by veterinary scientists and clinicians.

In horses, the ingestion of excessive amount of rapidly fermentable carbohydrates, for example, an overload of starch from cereal grains or sugar and fructans from pasture or the feeding of black walnut extract can reproducibly induce laminitis (85), a condition that has been associated with OS (6). Rapidly fermented carbohydrates induce lactic acid production, increase hindgut mucosal permeability and therefore endotoxins [lipopolysaccharides (LPS)], exotoxins (protease), and amines are released by the disturbed gut microflora leading to the activation of neutrophils, release of cytokines and other inflammatory and OS mediators, which ultimately lead to the activation of matrix metalloproteinase which results in laminitis (85). Evidence of OS has been reported in the pathophysiology of black walnut extract induced laminitis (86), and it has been thought that both lipid and protein oxidation might be more prominent in the carbohydrates overload model (87). However, while the results of Burns et al. (87) study did not show an increase in laminar protein carbonyl content in either the black walnut extract and carbohydrates overload induced laminitis, it could be argued that the sampling window adopted in that study was not able to capture the formation of these products or that some antioxidant mechanism might have been upregulated in these models (87), conferring some degree of protection of the laminar tissue. Finally, it has been reported that synovial fluid protein carbonyl content is increased in equine joints under degenerative process (88, 89). While the increase in protein oxidation could have been the consequence of a post traumatic inflammatory response, further studies are required to determine the overall contribution of OS to the pathophysiology of these diseases.

An increase in AOPP concentration has also been observed in dairy cows when they are fed maize silage (77, 90) and in growing dairy calves (91). Silage is characterized by low antioxidant content (92), and when its level in the diet is increased it could lead to OS. In addition, as the antioxidant capacity of rumen bacteria is less developed than that of aerobe microbes, the lower antioxidant content of the silage might also expose ruminal bacteria to OS, impairing their activity, growth, and finally decreasing ruminant production. Indeed, a negative correlation between AOPP concentration and milk yield has been observed in dairy cows (90). Interestingly, an increase in AOPP concentration has also been observed in obese ponies subjected to high level of energy restriction (93). This observation seems to be ascribable to the high level of lipomobilization as reflected by the increase in triglycerides and NEFA concentrations, which are then prone to oxidation. This observation further supports the link between energy balance, metabolism and oxidant-antioxidant balance (7, 8, 10). Finally, supplementing pigs’ diet l-methionine (l-Met) resulted in a reduction of MDA and protein carbonyl levels in duodenal mucosal samples, indicating a decrease in OS in the mucosa of the duodenum (94). It seems that this observation is due to the greater efficiency of l-Met, compared to dl-Met, in enhancing GSH synthesis in the intestinal mucosa. l-Met is also known to be a potent ROS scavenger (95); free l-Met and l-Met residues in protein act as endogenous antioxidants in cells, namely duodenal mucosal cells in Shen et al.’s (94) study.

Oxidative stress can be induced by dietary manipulation in poultry, for example, oxidized feeds can induce protein oxidation in birds (96). Indeed, feeding diets with oxidized oil (97) or animal-vegetable fat (98) increased plasma protein carbonyl content. Dietary antioxidant supplementation with organic selenium and minerals (97) or with saponins isolated from ginseng stems and leaves (99) is able to reduce OS in poultry by eliminating or decreasing the production of protein carbonyls. Therefore, we propose to include AOPP and protein carbonyl in the panel of biomarkers to study the relationships between OS, nutrition, metabolism, and gut health in veterinary medicine.

### Protein Oxidation and Mammary Gland Function

To the best of our knowledge, no thorough studies were undertaken to measure markers of protein oxidation in the periparturient cow, where a state of OS can be observed in response to the copious milk yield and mammary gland remodeling. At least three sources of OS can be identified: the shift in cellular metabolism during the transition period, the contribution of immune cells (PMN and phagocytes), and the intensive mammary epithelial cell replacement occurring after termination of milking. In a preliminary study, we observed that plasmatic AOPP significantly decreased and plasmatic carbonyl groups significantly increased in cows around parturition, suggesting that different radicals can produce different alteration in the protein structure and a panel of parameters may better characterize the OS status (100). However, further work is required to investigate the relationships between protein oxidation and mammary gland function.

Between two consecutive milking bouts most of milk produced resides within mammary alveoli and ducts for a significant time, exposing milk proteins to the action of oxidizing enzymes, such as MPO, lactoperoxidase, and xanthine oxidase, present in the surrounding mammary and immune cells (101). Thus, OS biomarkers in colostrum and milk may reflect the oxidative status within the mammary gland; and, perhaps, oxidized protein products in milk and whey can be non-invasive tools for investigating the oxidative status of the dairy cow. We are currently undertaking a series of experiments to investigate if biomarkers of oxidized protein can be measured in colostrum and milk, and to characterize differences in oxidized protein products potentially related to HOCl and SCC. Preliminary results suggested that exposure of milk to increasing HOCl concentrations (HOCl/protein ratio from 0.3 to 3 μmol/mg) induces a 20–30% increase in AOPP concentrations and a three to sixfold increase in carbonyl group concentrations (102). In addition, a significant correlation has been observed between milk AOPP and somatic cell count (100), further supporting the involvement of OS in mammary gland function. A deeper characterization of the role of protein oxidation during lactation might allow the development of targeted antioxidant supplementation that might optimize mammary gland health by favoring tissue repair and cell turnover and prevent diseases like mastitis.

### Protein Oxidation and Respiratory Diseases

The respiratory system is a major site of OS insult and it seems that pulmonary OS is crucial for the progression of respiratory disease especially in horses (103). It has been reported that AOPP concentration was numerically but not significantly elevated in foals affected by *Rhodococcus equi* (104). A possible explanation for the non-significant difference in AOPP in Crowley’s study is that neutrophil counts did not differ between healthy and affected foals. Considering that higher neutrophil counts have been observed in foals with manifest clinical signs of *R. equi* pneumonia (105), it is possible to speculate that AOPP concentrations may differ beyond the subclinical state. Indeed, the observations that AOPP values were greatly elevated in a foal showing overt evident of upper respiratory tract disease associated with a heavy nasal culture of *Streptococcus equi* suggest that this might be the case (106). In Crowley’s study, the AOPP/albumin ratio was significantly higher in *R. equi* affected foals compared to their healthy counterparts. This observation brings further evidence that AOPP/albumin ratio seems to be a more sensitive biomarker of OS suggesting that a more integrated approach to OS research and its role in the inflammatory events is required (107).

Lungs are quite susceptible to LPS, a major component of the outer membrane of gram-negative bacteria. Studies in piglets have shown that ampelopsin, a flavonoid with known antioxidant activity, is able to reduce the lung protein carbonyl content in LPS-challenged piglets (108). Recently in a study designed to evaluate OS responses in fetal lambs exposed to intra-amniotic endotoxin, a significant increase in protein carbonyls was observed in their bronchoalveolar lavage fluid (BALF) and in their plasma (109). Therefore, the investigation of proteins oxidation could represent a novel tool to study the role of OS in the etiopathogenesis of respiratory diseases in veterinary medicine.

### Protein Oxidation and Parasitic Infection

Microorganisms, like bacteria, viruses, and parasites, can increase ROS production inducing OS (6). Parasitic diseases induce inflammation resulting in an influx of eosinophils, which seems to be responsible for tissue damage, possibly via their potent ROS production and therefore exposing farm animals to OS (7). Gastrointestinal nematode infections are quite prevalent in grazing animals and it has been observed that the selenium status of sheep may influence the acquired immunity to parasitic infestation (110). For example, during *Strongyloides papillosus* infections, it has been observed that OS damage seems to be related to the severity of the parasitic infection, and that sheep were exposed to OS even after they were treated with albendazole (111). The observed decrease in albumin and total thiol groups, which was accompanied by an increase in urea and protein carbonyl groups in the study of Dimitrijević et al. (111), clearly indicates that protein oxidation plays an important role in the etiopathogenesis of parasitic infections. Of particular interest is the observation that the changes in protein oxidation markers were correlated with the intensity of parasitic infection, which not only brings further proof of an impaired oxidant/antioxidant balance, but it also suggests that changes in protein oxidation markers may reflect the extent of damage induced by parasites and potentially different stages of the parasitic infection.

Theileriosis is another parasitic disease caused by a small protozoan that infects both red and white blood cells. In cattle, *Theileria annulata* infection induces high levels of proinflammatory cytokines (112) and OS damage of erythrocytes’ skeletal membrane protein resulting in an increase in protein carbonyls (113). The observation that *T. annulata* infection in calves is associated with higher levels of protein oxidation than lipid oxidation suggests that protein carbonyls might be more reliable markers of OS damage during parasitic infection. Therefore, protein oxidation biomarkers could be used as monitoring tools aiding in diagnostic, prognosis, and anthelmintic therapeutic decision-making processes.

### Protein Oxidation and Husbandry Practices

Oxidative stress biomarkers have been proposed as new and reliable indicators of animal welfare since stress of any origin can deplete the body’s antioxidant resources (114). For example, an increase in plasma protein oxidation has been reported in pigs housed at high densities (115). Observations made in cattle kept in good or harsh conditions by the same research group have revealed that protein oxidation was highest in cattle living in hard conditions (116). While these observations could be due to several factors (nutrition, environmental conditions), they also suggest that animals might adopt different behavioral strategies in order to cope with stress (115). This observation highlights the need of a multidisciplinary approach to the characterization of OS-related diseases as both nutrition, metabolism, behavior and their interactions with potential pathogens might contribute to the exacerbation of OS.

### Protein Oxidation and Neonatal Physiology

There is much interest in the mechanisms by which OS may influence postnatal growth, metabolism, and health in neonatal animals (117). At birth, the passage from the intrauterine hypoxic environment to the relatively hyperoxic external environment induces an abrupt exposure of mammalian neonates to environmental oxygen. In response to the changes in extracellular environmental conditions, the cells of new-born animals generate large amount of reactive ROS leading to neonatal OS (117).

In a study designed to evaluate the oxidant/antioxidant balance in dairy calves from birth to weaning, it has been reported that antioxidative defenses increased with time in new-born calves and this process seemed to be related to protein oxidation (118). In particular, it was observed that plasma AOPP concentration and the AOPP/albumin ratio progressively decreased from birth to weaning, while the opposite trend was observed for albumin and thiol groups. Albumin not only is the predominant oxidized protein contributing to AOPP formation but also constitutes the largest pool of circulating thiols; indeed, a positive correlation between albumin and thiol groups has been reported (119). A negative correlation between plasma albumin and thiol groups concentration, and AOPP concentrations and with the AOPP/albumin ratio was also observed in Ranade et al. (118) study, bringing further supports to the role of albumin as the predominant plasma protein contributing to AOPP formation.

It has been reported that protein carbonyls concentration in the serum of fetal and neonatal pigs were quite similar, suggesting that the oxidative mechanisms that regulate the production and degradation of circulating protein carbonyls are similar *in utero* and during early post-uterine life (119). In their study, Caperna et al. (119) also compared protein carbonyls between normal and low birth-weight piglets finding no differences. While this observation needs to be further explored, an interesting finding from this study was that two potential specific upregulated carbonylated markers were observed in low birth-weight piglets at birth (119). In a study aimed at investigating OS and the development of an antioxidant system after early weaning in piglets, it has been observed that plasma protein carbonyl levels were increased from day 1 to day 5 after early weaning (120). While weaning can alter the oxidant/antioxidant balance in piglets, this balance seems to be restored with the development of an antioxidant system via feedback regulation (120). Although additional studies are needed to isolate and identify them, these findings suggest that the study protein oxidation markers might increase our knowledge of redox homeostasis during the neonatal period, allowing the development of specific antioxidant interventions.

### Protein Oxidation and Environmental Conditions

Heat stress has been implicated in the generation of OS either through excessive ROS production or decreased antioxidant defenses (121–123). Heat stress increases AOPP concentration in sheep and pigs and supra-physiological doses of Vitamin E and selenium are able to reduce the oxidation of plasma protein (124, 125). The lower AOPP concentration in heat stressed lambs fed supra-physiological doses of Vitamin E and selenium is indicative of protective role of these two antioxidants against the OS damage of proteins induced by heat stress and reinforces the need for higher levels of antioxidants than the current recommended levels under stressful conditions. Studies in broiler chickens have also demonstrated that high temperatures induce both lipid and protein oxidation (126); however, in another study aimed at assessing the effect of acute and chronic heat stress in broiler breeders, no changes in both lipid and protein oxidation were observed (127). While this apparent inconsistency could be ascribed to the different temperature regimes adopted in the two studies, it seems that acute heat stress might induce OS by disrupting the respiratory chain complex (126). Interestingly, low ambient temperature conditions increase the amount of protein carbonyls in liver and lung of broiler chickens; however, vitamin C dietary supplementation was not able to reverse this effect (128). Cold can be considered as a stressor and therefore lower temperature can induce OS.

The increase in protein oxidation following heat stress is of particular interest as it may have important implications on protein accretion and oxidative stability in lamb muscles, which is very important from meat quality perspectives for both ruminants and poultry (4, 96). The increase in AOPP during heat stress was accompanied by an increased expression of TNF-α in skeletal muscle in non-supplemented sheep (123), that further suggests a strong association between protein oxidation and mediators of proinflammatory responses (107, 129), and indicating that heat stress leads to undesired proinflammatory responses that may compromise animal performance (4). Maintaining animal performance during hot and humid weather requires continued advances in nutritional strategies to enhance animal resilience to heat stress. Therefore, the manipulation of dietary micronutrients and antioxidants has the potential to prevent the effects of oxidative damage (4, 130). Considering the capacity for interactions of antioxidants with other dietary substrates and environmental conditions, such as heat stress (131), it is possible that a more sophisticated understandings of the mechanisms responsible for the impairment of redox homeostasis induced by heat stress may help to generate mitigation strategies (132) and to develop potential nutritional interventions to alleviate the negative consequences of heat stress (133).

## Conclusion

For a better understanding of the role of OS and protein oxidation in veterinary medicine, future studies should identify a panel of biomarker of protein oxidation to be used in veterinary medicine. While an OS biomarker should meet a number of criteria (Table 2), it is clear then that each biomarker of protein oxidation has its benefits and shortcomings. The biomarkers that have been used so far, while it could be argued that some of them could be considered too generic, they have been useful in the characterization of pathophysiology of protein oxidation. In our opinion, the translation of the current knowledge into practical applications for veterinary scientists and clinicians needs to be made a priority by the scientific community as this would allow to achieve a better insight in the evaluation and in the progression of several conditions and associated therapeutic interventions. Future studies should be combined with a proteomic analysis approach so that we can identify the specific sites of oxidation damage on particular proteins allowing us to refine the panel of protein oxidation biomarkers to be used in the characterization of OS induced conditions that lead to decreased welfare and productive performances.

**Table 2:** **Key criteria of the validation process of a suitable biomarker of protein oxidation**.

Selection of biomarker of protein oxidation
Directly implicated in the onset and progression of disease
Development and validation of method for measuring the biomarker
Verification of potential for pitfalls and artifacts
Verification of biomarker in suitable animal models
Identification of modifying factor of the biomarker (nutrition, physiological status, and photoperiod)
Establishment of reference intervals and values
Adaptation of the method to field condition (sensitivity, simplicity, and throughput)

**Figure 2:**
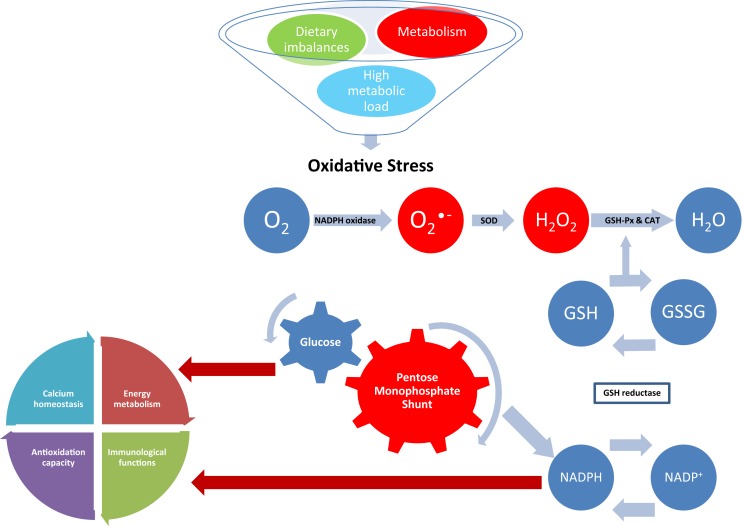
**Effect of oxidative stress on NADPH-dependent metabolic pathways**. Superoxide $\left({\text{O}}_{2}^{•-}\right)$ is generated during normal metabolism by the activity of the NADPH oxidase. Dietary imbalances and high metabolic load also stimulate the activity of the NADPH oxidase leading to oxidative stress if not removed by the antioxidant system. In normal conditions, ${\text{O}}_{2}^{•-}$ is converted in hydrogen peroxide (H_2_O_2_) by superoxide dismutase (SOD); H_2_O_2_ is then converted in water (H_2_O) by the activity of glutathione peroxidase (GSH-Px) and catalase (CAT). Reduction of peroxides is accompanied by oxidation of reduced glutathione (GSH), which can be regenerated from glutathione disulfide (GSSG) by reducing equivalents from NADPH, which is generated by the pentose monophosphate shunt. The resulting destruction of GSH increases consumption of reducing equivalents, diverting glucose from important physiological pathways and competing with NADPH-dependent metabolic pathways, such as energy metabolism, immunological functions, antioxidation capacity, and calcium homeostasis.

This review has highlighted how considerable evidence on the involvement of OS and protein oxidation in a number of diseased and conditions of farm animals has been obtained in the past few years. These studies span from important physiological functions, such as reproduction, nutrition, metabolism, lactation, gut health, and neonatal physiology, to respiratory diseases and parasitic infection. Despite this body of evidence, there are several questions that need to be explored about the role that OS and protein oxidation play in animal health. Animal health and welfare is crucial for a sustainable production system, and the characterization of the mechanisms by which OS may influence metabolism and health is now attracting international interest. The study of oxidant/antioxidant balance is contributing to the understanding of important pathways involved in regulation of cellular functions and metabolism. When ROS accumulates because of high metabolic rates, this may result in an impairment of the redox balance leading to OS (Figure 2). Therefore, as OS can negatively impact immune function and associated health disorders, future research should investigate the mechanisms involved in the maintenance of redox homeostasis especially during particular physiological conditions when animal metabolism is significantly increased, and homeostatic and homeorethic mechanisms are challenged.

## Conflict of Interest Statement

The authors declare that the research was conducted in the absence of any commercial or financial relationships that could be construed as a potential conflict of interest.
